# Hippocampal neuroimmune response in mice undergoing serial daily torpor induced by calorie restriction

**DOI:** 10.3389/fnana.2024.1334206

**Published:** 2024-04-15

**Authors:** Valeria Cogut, Maaike Goris, Aukje Jansma, Marrit van der Staaij, Robert H. Henning

**Affiliations:** Department of Clinical Pharmacy and Pharmacology, University Medical Center Groningen, Groningen, Netherlands

**Keywords:** laboratory mouse, daily torpor, calorie restriction, neuroinflammation, microglia morphology

## Abstract

Hibernating animals demonstrate a remarkable ability to withstand extreme physiological brain changes without triggering adverse neuroinflammatory responses. While hibernators may offer valuable insights into the neuroprotective mechanisms inherent to hibernation, studies using such species are constrained by the limited availability of molecular tools. Laboratory mice may serve as an alternative, entering states of hypometabolism and hypothermia similar to the torpor observed in hibernation when faced with energy shortage. Notably, prolonged calorie restriction (CR) induces serial daily torpor patterns in mice, comparable to species that utilize daily hibernation. Here, we examined the neuroinflammatory response in the hippocampus of male C57BL/6 mice undergoing serial daily torpor induced by a 30% CR for 4 weeks. During daily torpor episodes, CR mice exhibited transient increases in TNF-α mRNA expression, which normalized upon arousal. Concurrently, the CA1 region of the hippocampus showed persistent morphological changes in microglia, characterized by reduced cell branching, decreased cell complexity and altered shape. Importantly, these morphological changes were not accompanied by evident signs of astrogliosis or oxidative stress, typically associated with detrimental neuroinflammation. Collectively, the adaptive nature of the brain’s inflammatory response to CR-induced torpor in mice parallels observations in hibernators, highlighting its value for studying the mechanisms of brain resilience during torpor. Such insights could pave the way for novel therapeutic interventions in stroke and neurodegenerative disorders in humans.

## Introduction

Hibernation is a unique adaptation enabling certain species to endure harsh conditions, such as extreme temperatures and food shortages (Ruf and Geiser, 2015). It consists of torpor bouts, where metabolic rate and body temperature are drastically reduced for weeks to months in deep hibernators, alternated with short arousals lasting less than 24 h, during which normal physiological functions resume (Carey et al., 2003; Geiser, 2004). Remarkably, torpid deep hibernators endure drops in cerebral blood flow to as low as 10% of the normal value and subsequent reperfusion during arousal without noticeable neuronal damage (Frerichs et al., 1994; Frerichs and Hallenbeck, 1998; Ma et al., 2005). Non-hibernating mammals cannot withstand such changes and are particularly vulnerable to reperfusion injury (Yang and Betz, 1994; Aronowski et al., 1997; Dave et al., 2006; Bogren et al., 2014; Bogren and Drew, 2014), a complex cascade of inflammatory and oxidative processes leading to neuronal death and brain damage (Hallenbeck et al., 1986; Barone et al., 1992; Aronowski et al., 1997; Kuroda and Siesjo, 1997; Love, 1999). While it is unknown how hibernators evade this injury, a series of coordinated adaptations are believed to underlie this natural tolerance (Dave et al., 2012). Apart from reduction in cerebral blood flow, deep torpor poses several additional challenges to the brain including widespread hyperphosphorylation of the microtubule-associated protein tau (Arendt et al., 2003), synapse reorganization and spine retraction (Popov and Bocharova, 1992; Popov et al., 2007), and pronounced changes in microglia morphology (Cogut et al., 2018; Leon-Espinosa et al., 2018). Yet, these physiological changes are rapidly and completely reversed during arousals.

Unlike deep hibernators, smaller species mostly adopt a daily torpor strategy, with torpor bouts lasting between ~3 and 12 h, alternated by euthermic periods throughout the day. Daily torpor bouts are characterized by a milder reduction in metabolism and body temperatures between 15 and 30°C (Ruf and Geiser, 2015). To date, knowledge of torpor-associated brain mechanisms in daily torpor is even more scarce. Djungarian hamster brain features a reversible hyperphosphorylation of the tau protein during daily torpor similar to deep hibernators (Boerema et al., 2008). Further, transcriptomics studies in its hypothalamus have identified gene expression changes analogous to those reported in deep hibernators (Cubuk et al., 2017; Haugg et al., 2022). These findings suggest that torpor-associated molecular changes of deep and daily hibernators are (partly) overlapping. While daily torpor offers a practical model to study these brain changes, the variability between species and even among individuals of the same species poses significant challenges (Ruf and Geiser, 2015).

The laboratory mouse (*Mus musculus*) is a valuable species for studying torpor’s brain effects due to its cost-effectiveness, ease of genetic manipulation, and a fully annotated genome (Rydell-Tormanen and Johnson, 2019). Mice undergo daily torpor when faced with energy imbalances from limited food and colder environments (Hudson and Scott, 1979; Gavrilova et al., 1999; Hut et al., 2011; Jensen et al., 2013). One method to induce torpor is through moderate caloric restriction (CR) (Himms-Hagen, 1985; Walford and Spindler, 1997; Mitchell et al., 2015). When subjected to a prolonged calorie reduction of 30–40%, mice progressively undergo more frequent and deeper torpor bouts, reaching body temperatures below 25°C for several h (Mitchell et al., 2015). Importantly, the serial daily torpor patterns that emerge closely resemble the natural torpor patterns observed in Djungarian hamsters (Ruf and Heldmaier, 1992), providing a robust model for studying torpor’s effects on the brain and underlying mechanisms.

In this study, we induced serial daily torpor in male C57BL/6 mice subjected to a 30% CR for 4 weeks and assessed the oxidative stress and neuroinflammatory response in the hippocampus during various stages of torpor and arousal. Our findings reveal a phase-specific increase in mRNA expression of inflammation-associated genes and persistent microglia morphological alterations. However, in the absence of pronounced oxidative stress and evident gliosis, our data suggest that these changes represent neuroimmune adaptations to daily torpor, similar to those observed in the deep hibernator Syrian hamster (Cogut et al., 2018; Leon-Espinosa et al., 2018).

## Methods

### Animals

Male C57Bl/6J mice were bred in-house at the Groningen Animal Research Center (CDP). Prior to experiments, mice were group housed at 22°C under a standard 12:12 light–dark cycle, with *ad libitum* access to water and standard lab chow. Experimental procedures were conducted under the approval of the Animal Welfare Body (IVD) of the University of Groningen after approval of the competent authority [Dutch Central Committee for Animal Experiments (CCD), permit AVD105002016427].

### Daily torpor paradigm

Animals were 4 months ±2 weeks of age at the start of the experiment. One week prior to the start of caloric restriction (CR), all mice were single-housed in a designated quiet room at an ambient temperature of 21°C under a 12:12 light–dark cycle, with lights on at 7:00 AM. Caloric intake was reduced to 70% of their individual food intake at their initial body mass, with a 10% calorie reduction every 2 days. CR mice received food at zeitgeber time (ZT) 8 and typically consumed it within 3 h, before the lights turned off. Control mice received 120% of their daily caloric intake at ZT + 8 and remained euthermic (EU) without entering torpor. CR mice exhibited a shift in their circadian activity patterns, becoming diurnal instead of their typical nocturnal behavior, as previously described (van der Vinne et al., 2014). After a subsequent CR for 3 weeks, mice were housed in calorimetric cages (TSE, Bad Homburg, Germany) to measure metabolic rates (VO_2_ in mL/h), CO_2_ production, and to calculate the respiratory exchange rate (RER). Based on VO_2_ consumption, mice were euthanized at different timepoints in their daily torpor cycle. Pre-torpor (PT) mice were sacrificed at ~ZT15, 15 h post-arousal and just before the expected onset of hypothermia and hypometabolism, when still active and with a body temperature (Tb) above 36°C. Torpor late (TL) mice were sacrificed at ~ZT23, approximately 6 h into torpor. Arousal early (A1) mice were sacrificed at ~ZT1, 1 h into arousal showing increased motor function and awareness but not yet fully active. Arousal late (A8) mice were sacrificed at ~ZT8, 8 h after arousal onset, by which time they were fully active and euthermic. Control EU mice were sacrificed at the same time as the A8 group, both prior to feeding. Additionally, a refed group (RF), receiving excess food over the last 2 days to interrupt torpor, was sacrificed at the same time as PT mice.

### Tissue collection and preparation

Mice were anesthetized in a darkened inhalation box containing 5% isoflurane in oxygen/air (30/70%) before being moved to the operation room. Rectal temperatures were taken approximately 3 min after anesthesia induction by inserting the thermocouple approximately 2 cm into the mouse’s rectum and waiting for 30 s. For qRT-PCR and Western Blot, animals (*n* = 57) were transcardially perfused with 0.1 M PBS after which the hippocampus was dissected, snap-frozen in liquid nitrogen, and stored at −80°C. For immunohistochemical (IHC) staining, animals (*n* = 20) were transcardially perfused with 0.1 M PBS, followed by 4% paraformaldehyde (PFA) in 0.1 M PBS. Whole brains were dissected, post-fixed in PFA for 24 h at 4°C, dehydrated with 30% sucrose in PBS overnight at room temperature (RT) and subsequently frozen at −50°C and stored at −80°C.

### RNA isolation and RT-qPCR

RNA from hippocampus was extracted using RNeasy Lipid Tissue Mini kit (Qiagen #74804). After RNA isolation, RNA was reversed transcribed and quantitative PCR was performed as previously described (Cogut et al., 2018). Primers used are listed in Supplementary Table 1.

### Western blot

Protein lysates from hippocampus were obtained using RIPA lysis buffer (50 mM Tris-Cl pH 8.0, 150 mM NaCl, 1% Igepal Ca-630, 0.5% sodium deoxycholate, 1.0% sodium dodecyl sulfate) enriched with 0.4% protein inhibitor cocktail (Roche Diagnostics #11836170001), 1 mM sodium orthovanadate (Sigma-Aldrich #S6508), 10 mM NaF, 10 mM β-mercaptoethanol (Sigma-Aldrich #805740). Total protein concentrations were measured with Bradford assay (Bio-Rad Laboratories #5000116) and the samples were loaded onto 4–20% sodium dodecyl sulfate-polyacrylamide pre-casted gels (Bio-Rad TGX gels #456–8095). Western Blotting was performed as previously described (Nakladal et al., 2022). Membranes were probed with anti-4 hydroxynonenal antibody (1:1000 dilution, Abcam #ab46545). Chemiluminescent signal was normalized to total protein loading using the standard BioRad StainFree TGX blot technology. Analysis was performed in ImageLab 6.0.1 (Bio-Rad).

### Immunohistochemistry, image acquisition and quantification

For immunofluorescence analysis, five free-floating coronal brain sections (25 μm) from the dorsal hippocampus (bregma coordinates −1.6 mm to −2.1 mm) were collected per animal and transferred to a 24-well plate. Slices were permeabilized with 0.5% Triton X-100 (Sigma #T8787) in 0.5% TBST (0.5% Tween-20 in 1xTBS) for 1 h at RT and then blocked in 5% bovine serum albumin (BSA, Westburg #ab270701) for 30 min at RT. Sections were incubated with primary antibodies diluted in 0.1% Triton X-100, 2.5% BSA, 1XTBST at 4°C overnight, followed by washing and incubation with respective secondary antibodies for 2 h at RT. Primary antibodies used were anti-GFAP (1:1000, chicken, Novus #3NBP1-05198) and anti-IBA-1 (1:1000, rabbit, Wako #019-19741). Secondary antibodies used were Alexa 488 anti-rabbit (1:500, Invitrogen #A-11008) and Alexa 594 anti-chicken (1:500, Invitrogen #A-11042). The sections were finally mounted on SuperFrostPlus Slides (Invitrogen) using Dapi containing Vectashield mounting media (Vector laboratories #H-1200-10) and imaged on a Zeiss LSM 780 confocal system. Z-stacked photomicrographs from pre-defined regions of interest were acquired using a 20× (for GFAP) and 40x (for IBA-1) objective lenses. Consistency in microscope settings and Z-stack parameters was maintained across all samples. GFAP fluorescence intensity from at least five photomicrographs per animal was quantified as the mean gray value in Fiji software (Schindelin et al., 2012).

IBA-1-positive microglia morphology was assessed using the AnalyzeSkeleton and FracLac plugins in Fiji according to the previously published protocol (Morrison and Filosa, 2013; Young and Morrison, 2018). For each animal, 15 photomicrographs from three sections were selected. Images with unclear microglial cell bodies due to staining or imaging artifacts were excluded. Microglia ramification was first assessed using the AnalyzeSkeleton plugin. In brief, photomicrographs were converted to 8-bit images and the FFT bandpass filter was applied. The brightness and contrast were adjusted to allow optimal visualization of microglial branches. The unsharp mask was then applied to further enhance the image contrast, and the despeckle function was employed to eliminate resultant noise. The image was binarized, followed by despeckle, close, and remove outliers functions to remove single-pixel background noise and gaps between processes. The image was then skeletonized and further processed using the AnalyzeSkeleton (2D/3D) function. Cell somas were manually counted to determine the total microglial count per image. Measurements of total microglial branch length and number of branch endpoints were normalized to the number of microglial cells in each frame. Next, microglia underwent fractal analysis. Briefly, using the binarized images from the skeleton analysis, a region of interest (ROI) was made with the rectangle tool to fit all microglial cells. Using the ROI, 2–3 microglial cells per image were randomly selected, averaging 24 cells per animal. The selected cells were individually processed. The paintbrush tool was used to remove adjacent cell processes and isolate the cell of interest, and connect fragmented processes using the original photomicrograph as a reference. The binary image was then converted to an outline. The cells were subsequently analyzed with the FracLac plugin by selecting the “box counting” method and setting the “grid design Num G” to 4. The convex hull and bounding circle of each cell were calculated. The fractal dimension (a measure of shape complexity), the convex hull area and perimeter (indicators of the overall territory area of the cell), and the convex hull span ratio and circularity (reflecting cell shape) were measured.

### Statistics

All statistical analysis was performed in GraphPad Prism (version 9, GraphPad Software Inc., San Diego, CA, USA). Differences between the groups were compared using one-way ANOVA with Tukey’s post-hoc for multiple comparisons for normally distributed data. For data non-normally distributed, statistical analyses were performed using the Kruskal-Wallis test. The Pearson correlation coefficient was used to assess the linear relationship between variables. Statistical significance was defined as *p < 0.05, **p < 0.01, ***p < 0.001, ****p < 0.0001. Investigators were blinded during the molecular analysis.

## Results

### Serial daily torpor induction in mice by calorie restriction

**Figure 1:**
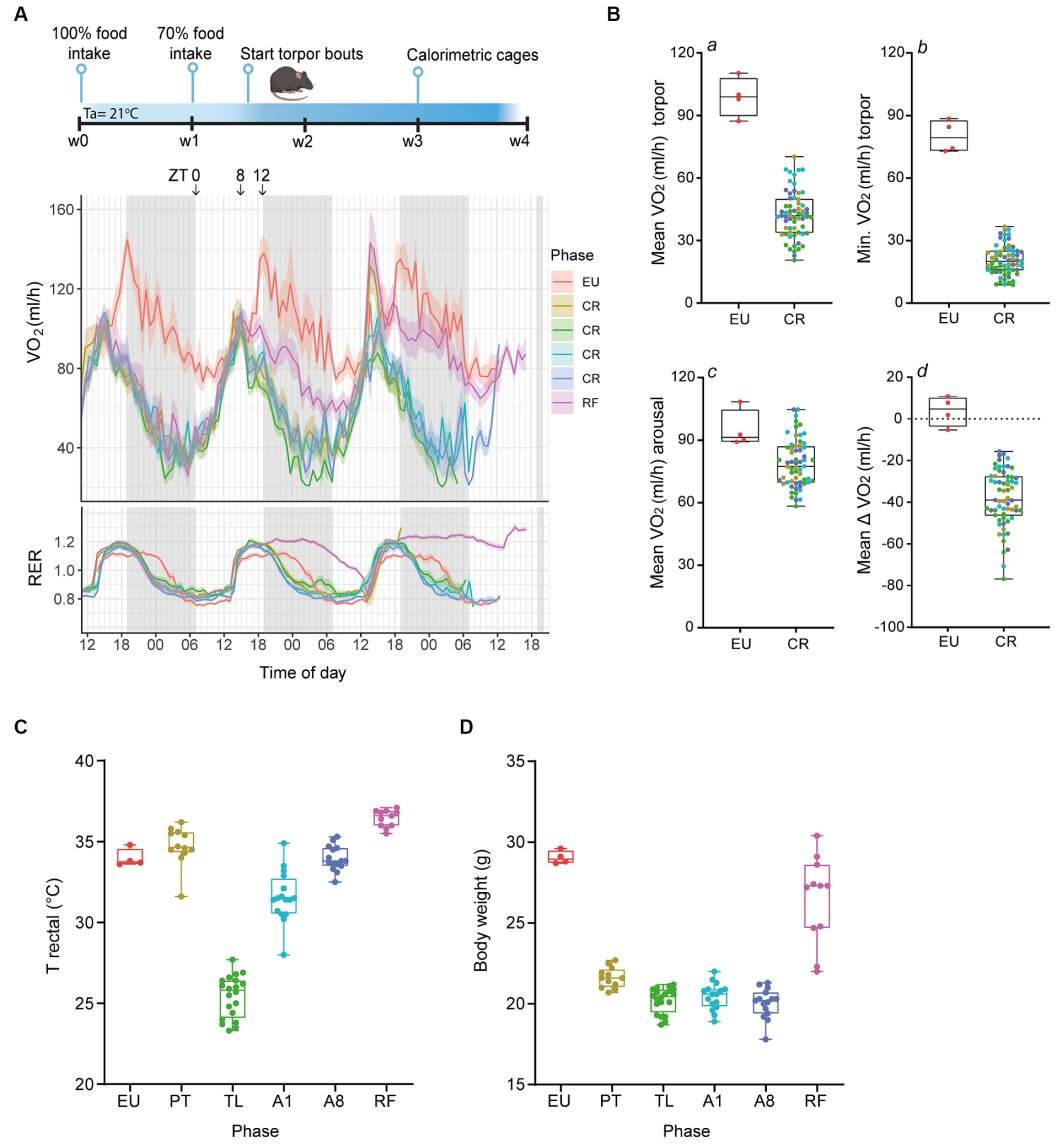
Serial daily torpor induction in mice by calorie restriction. **(A)** Schematic outline of the experimental timeline and representative calorimetric data from the last three consecutive days depicting the oxygen consumption rates (VO_2_, in mL/h) and respiratory exchange ratios (RER) for non-restricted (EU), calorie-restricted (CR), and refed (RF) mice. **(B)** Quantification of VO_2_ consumption during torpor (a, b), arousal (c), and the mean change in VO_2_ consumption between these phases (d) for EU and CR groups. **(C,D)** Rectal body temperatures and body weights at termination for control EU mice and CR- PT, TL, A1, A8 groups and RF mice. EU, euthermia; PT, pre-torpor; TL, torpor late; A1, arousal early; A8, arousal late. Data are presented as boxplots, where the central line indicates the median, the box edges represent the interquartile range (IQR) and the whiskers extend up to 1.5 times the IQR.

Torpor in mice was induced by gradually reducing calorie intake to 70% of the normal intake and then maintained for 3 weeks with timed feeding at ZT + 8. Control euthermic mice (EU) were kept on food *ad libitum* with feeding at ZT + 8 and did not enter torpor. In addition, we included a refed group (RF) where mice were switched from CR to timed feeding with an excess of food for 2 days, essentially ending daily torpor. During the final week of CR, mice were placed into metabolic cages to measure VO_2_ consumption and CO_2_ production, and calculate RER (Figure 1A). Torpor bouts, characterized by a marked reduction in VO_2_ consumption rate, were consistently observed in all CR mice during the second half of the dark phase and early light phase, followed by an arousal period. The RF group showed an intermediate pattern of torpor during the first day of refeeding and recovered to the normal pattern of control EU by the second day.

Compared to control EU mice, where VO_2_ consumption rates consistently stayed above 80 mL/h, CR mice experienced a marked decline in metabolic rate, evidenced by an average VO_2_ of 42.5 ± 1.4 mL/h and reaching a minimum value of 20.7 ± 0.9 mL/h (Figure 1B, panel a, b). These effect sizes were consistent with previous findings from fasting-induced torpor in mice (Hitrec et al., 2019; Hrvatin et al., 2020; de Veij Mestdagh et al., 2021). The arousal phase in CR mice was marked by an increase in VO_2_ consumption rates to an average of 80.6 ± 1.8 mL/h, marginally below the control EU group levels (Figure 1B, panel c). Notably, the average VO_2_ consumption change between arousal and torpor phases was −38.1 ± 1.7 mL/h for CR mice, in contrast to a minor fluctuation in the control EU group (Figure 1B, panel d).

As described in detail in the methods section, animals were euthanized at different phases of daily torpor and the presence or absence of torpor and termination in the correct phases was corroborated by rectal temperatures (Figure 1C). By the end of the protocol, CR mice had lost ~30% of their initial body weight, with weights stabilizing at ~20 g (Figure 1D).

Together, these data demonstrate that 30% CR in mice yielded a highly reproducible daily torpor, which was quickly ended by timed feeding of excess food.

### Hippocampal 4-HNE protein levels remain unchanged in mice undergoing CR-induced serial daily torpor

**Figure 2:**
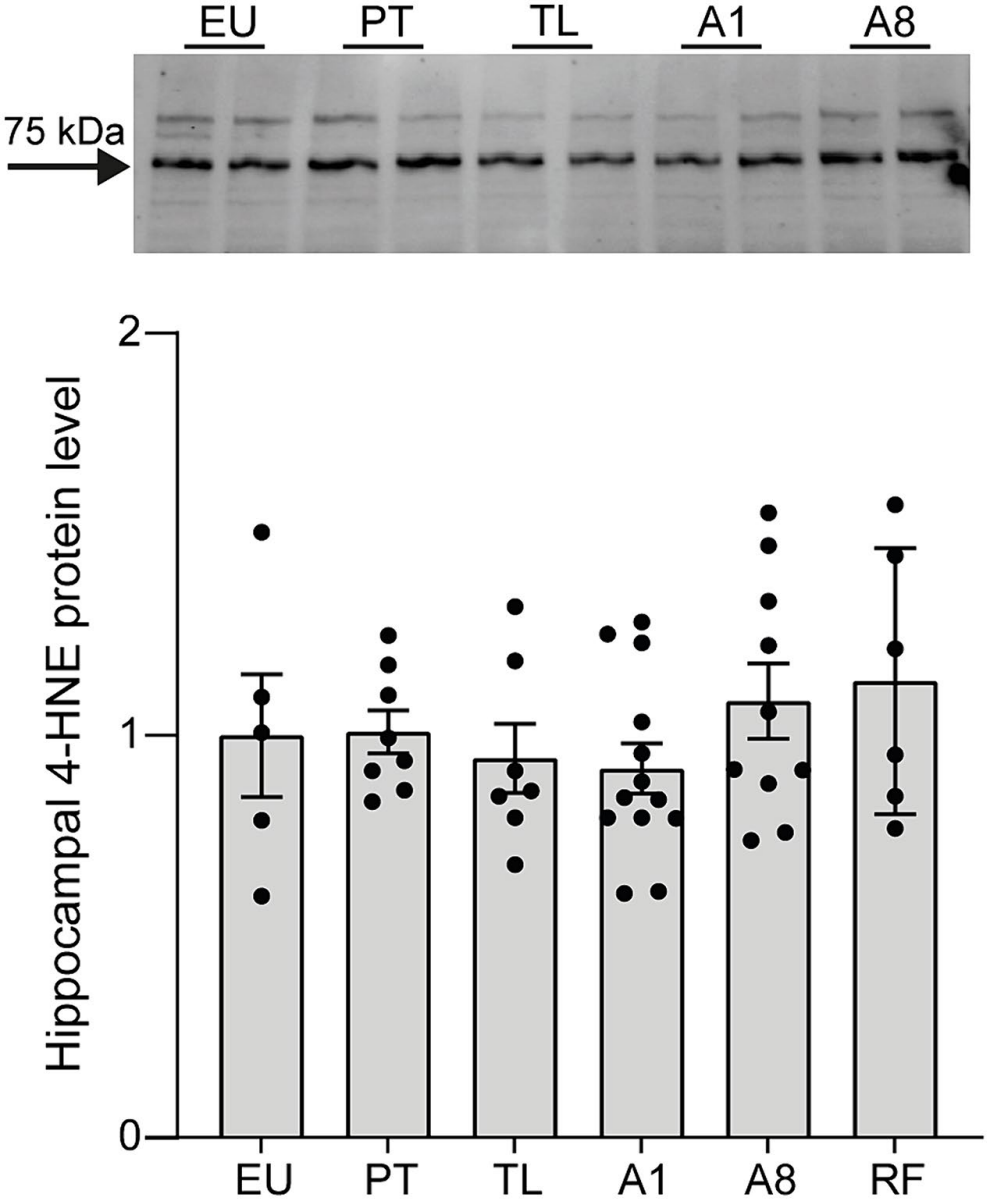
4-HNE protein expression levels remain unchanged throughout daily torpor in the hippocampus of CR mice. Representative western-blot analysis and protein expression quantification for control EU mice (not restricted, no torpor), calorie-restricted (CR) mice across torpor (TL) and arousal (A1, A8, PT) phases and refed (RF) mice. Expression was normalized to total protein (TPN) and expressed as a fraction of EU group. EU, euthermia; PT, pre-torpor; TL, torpor late; A1, arousal early; A8, arousal late. Data are mean ± SEM; *p* = ns.

We first assessed whether CR-induced serial daily torpor in mice leads to increased oxidative stress in the hippocampus, one of the brain regions especially vulnerable to oxidative damage (Wang and Michaelis, 2010). In non-hibernating mammals, oxidative stress is one of the major contributors to brain damage following ischemic events. Especially during reperfusion, the excess production of reactive oxygen species (ROS) can lead to lipid peroxidation, inflammation, and ultimately cell death (Chan, 2001). To this end, we quantified the protein levels of 4-hydroxy-2-nonenal (4-HNE), a byproduct of lipoperoxidation (Dalleau et al., 2013). Across all daily torpor phases (PT, TL, A1 and A8) and in RF mice, 4-HNE expression levels remained consistent and were comparable to the control EU group (Figure 2). These data suggest that the repeated torpor and arousal cycles in CR mice do not appear to result in increased oxidative stress of the hippocampus.

### TNF-α mRNA levels are transiently elevated in the hippocampus during daily torpor in CR mice

**Figure 3:**
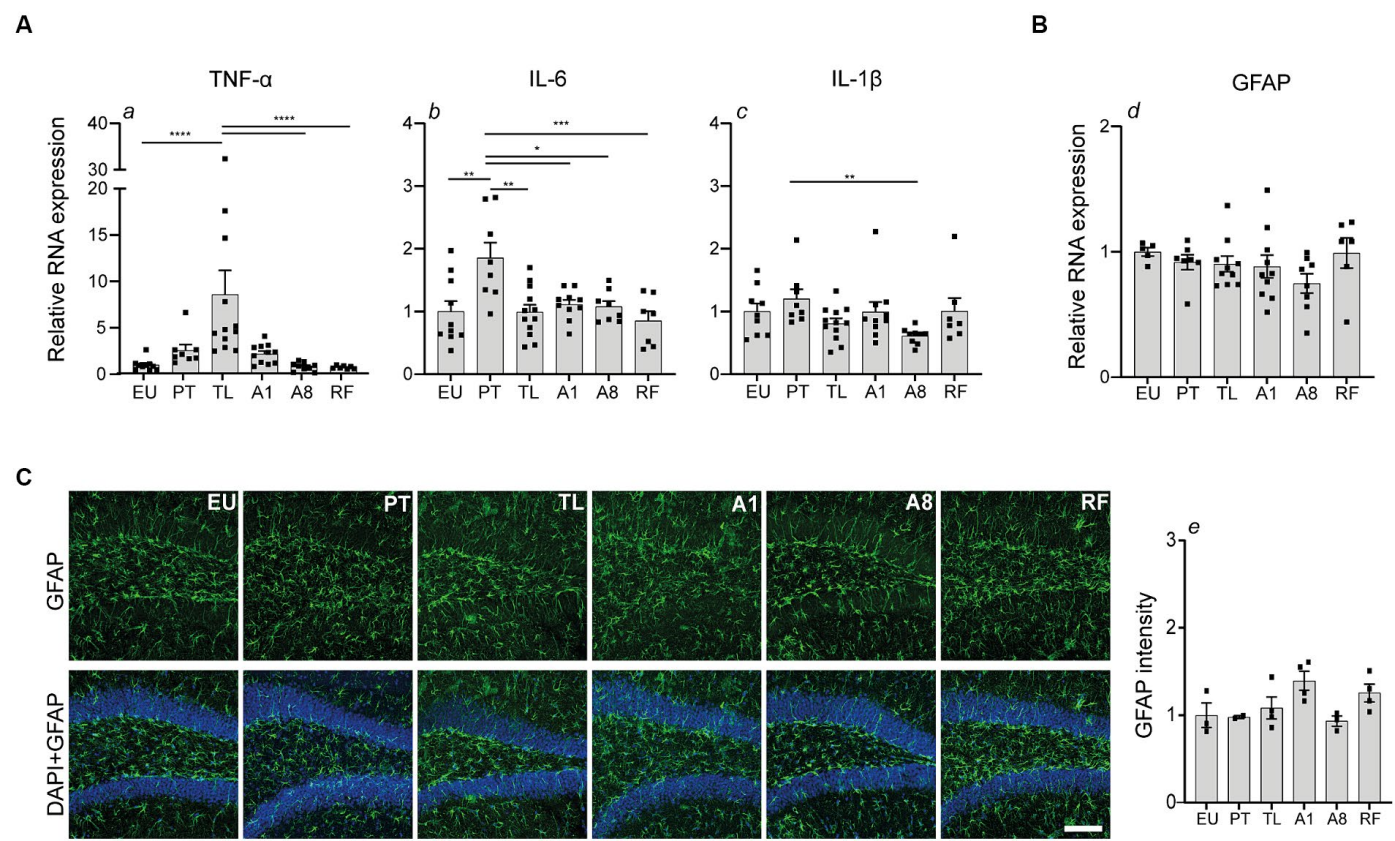
Transient elevation of TNF-α and IL-6 mRNA expression in the hippocampus during daily torpor in CR mice without evident astrogliosis. **(A)** Relative RNA expression of pro-inflammatory markers TNF-α, IL-6, IL-1β and **(B)** astrocyte marker GFAP in control EU mice (not restricted, no torpor), calorie-restricted (CR) mice across torpor (TL) and arousal (A1, A8, PT) phases and refed (RF) mice. RNA expression was normalized to Hmbs and expressed as a fraction of EU group. **(C)** Photomicrographs of GFAP staining in the dentate gyrus region of hippocampus. One representative full 25 μm Z projection is shown. Coronal brain sections were scanned using the confocal microscope at 20x magnification using the same acquisition settings between different groups. Immunofluorescent labeling for GFAP (green) and nuclear staining with DAPI (blue) is depicted. Quantification of GFAP fluorescence intensity was performed using Fiji software; *n* = 3–4 animals per group (except for PT group where *n* = 2 due to technical problems). Scale bar = 100 μm. EU, euthermia; PT, pre-torpor; TL, torpor late; A1, arousal early; A8, arousal late. Statistical analysis was performed with non-parametric Kruskal-Wallis test with Dunn’s post-hoc comparison (panels a, c, d, e) and one-way ANOVA test with Tukey post-hoc comparison (panel b). Data are mean ± SEM; * ≤ 0.05, ** ≤ 0.01, *** ≤ 0.001, **** ≤ 0.0001.

Next, we investigated whether CR-induced serial daily torpor initiates a neuroinflammatory response. We first examined the hippocampal mRNA expression levels of pro-inflammatory markers *TNF-α, IL-6* and *IL-1β* (Figure 3A). TNF-α expression was highly upregulated compared to the control EU group and showed the largest change throughout the torpor (TL) and arousal phases (A1, A8, PT). TNF-α expression peaked in TL, decreased by A1, and normalized to EU levels by A8. A slight, non-significant increase was observed in PT compared to A8 and EU. This trend was abolished by cessation of torpor (RF group). IL-6 mRNA levels were moderately increased in PT, but by TL, A1 and A8 phases, its levels were consistent with the control EU group. In contrast to TNF-α and IL-6, IL-1β exhibited stable mRNA expression levels comparable to the control EU group throughout all daily torpor phases.

Thereafter, we explored the impact on astrocyte reactivity using *GFAP*, a common marker of astroglial activation and gliosis. Quantitative PCR analysis showed no significant changes in GFAP mRNA expression across the torpor and arousal phases compared to the control EU group (Figure 3B). Consistent with the mRNA data, GFAP fluorescence in the dentate gyrus region of the hippocampus showed no major differences between the groups (Figure 3C).

Collectively, our findings indicate that CR-induced serial daily torpor in mice results in a transient, phase-specific upregulation of pro-inflammatory genes TNF-α and IL-6 during torpor and pre-torpor, respectively. Meanwhile, astrocyte activation remains largely unaffected as indicated by unchanged GFAP abundance.

### Hippocampal microglia undergo morphological alterations during daily torpor in CR mice

**Figure 4:**
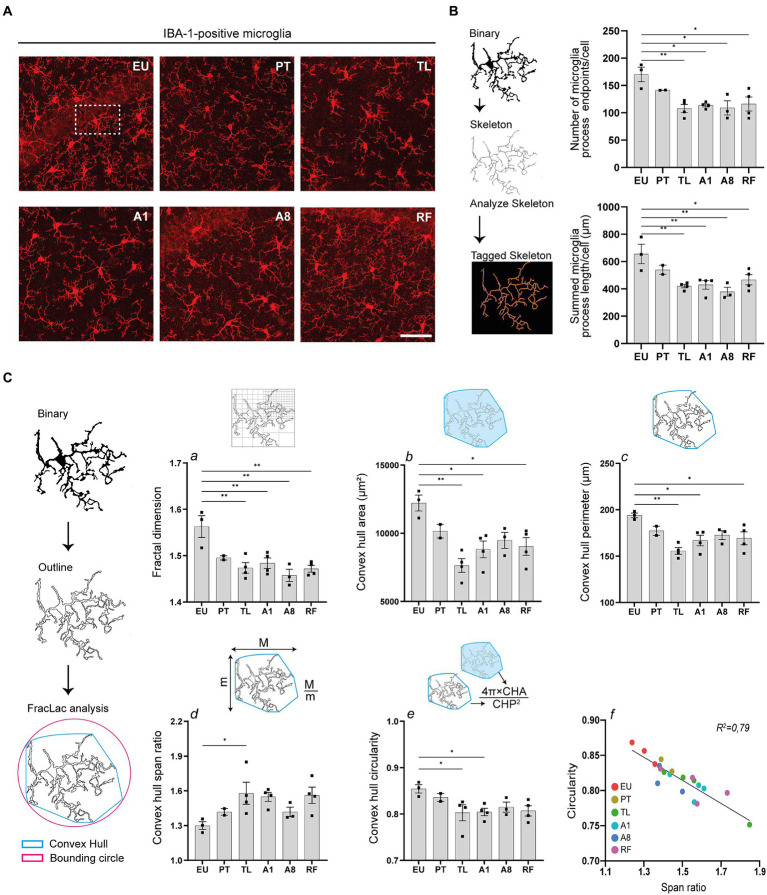
Hippocampal microglia undergo morphological changes during daily torpor in CR mice. **(A)** Photomicrographs of IBA-1 staining in the CA1 region of hippocampus in control EU mice (not restricted, no torpor), calorie-restricted (CR) mice across torpor (TL) and arousal (A1, A8, PT) phases and refed (RF) mice. One representative full 25 μm Z projection is shown. Coronal brain sections were scanned using the confocal microscope at 40x magnification using the same acquisition settings between different groups. **(B)** Maximum intensity projections of confocal images were converted to binary images and then skeletonized (for clarity, only one microglial cell is depicted). The number of microglia process endpoints (blue) and process length (orange) were normalized by the total number of microglia in each image. **(C)** Individual microglia were randomly selected from binarized images in **(B)** and processed for FracLac analysis. Fractal dimensions (a), convex hull area and perimeter (b, c), and convex hull span ratio and circularity (d, e) were measured. *N* = 3–4 animals per group (except for PT group where *n* = 2 due to technical problems). Scale bar = 50 μm. CA1, cornu ammonis 1 area; EU, euthermia; PT, pre-torpor; TL, torpor late; A1, arousal early; A8, arousal late. Statistical analysis was performed with one-way ANOVA test with Tukey post-hoc comparison and Pearson’s correlation test (**C**, panel f). Data are mean ± SEM; * ≤ 0.05, ** ≤ 0.01.

To further examine the neuroinflammatory response, we analyzed the morphology of microglia, the CNS’s resident macrophages, within the CA1 area of the hippocampus using Iba-1 immunohistochemistry to identify cells. Microglia cell ramifications decreased in PT, TL, A1 and A8 relative to the control EU group, with only partial reversibility in RF (Figure 4A).

We objectivated changes in microglia ramification using the AnalyzeSkeleton plugin in Fiji (Figure 4B). After binarizing and skeletonizing the dendritic branching of all microglia in each photomicrograph, process endpoints (blue) and process length (orange) of the tagged skeletons were quantified (Young and Morrison, 2018). The number of microglia process endpoints and the summed process lengths were significantly reduced during TL, A1, and A8 compared to the control EU group. Interestingly, even after cessation of torpor by refeeding (RF), reduced branching of microglia persisted.

Additionally, using the FracLac plugin, we observed significant changes in microglia complexity, size and shape (Figure 4C). After generating convex hulls and bounding circles of individual cells, we quantified several fractal parameters. Fractal dimensions, indicative of microglia complexity, significantly decreased during TL, A1, and A8 phases compared to the control EU group, which also persisted after ending of torpor by refeeding (RF) (Figure 4C, panel a). Concurrently, the convex hull area and convex hull perimeter, parameters reflecting microglia cell size, were also decreased. The TL phase showed the most significant decrease, with notable reductions also observed in the A1 phase, which persisted after cessation of torpor after refeeding (Figure 4C, panel b, c).

Alterations in microglia shape were also evident from the convex hull span ratio and convex hull circularity (Figure 4C, panel d, e). Span ratio, representing the elongation or aspect ratio of a cell, increased during TL and A1, suggesting a more elongated morphology during these phases. In contrast, the span ratios in A8 and PT more closely resembled those of the control EU group. The convex hull circularity, which indicates the roundness of the overall cell shape, significantly decreased during TL and A1 (Figure 4C, panel e). Expectedly, there was a strong negative correlation between the convex hull span ratio and circularity, implicating that elongated microglia indeed are to be less spherical (Figure 4C, panel f).

Collectively, the consistent decrease in microglia ramification and complexity, coupled with shape alterations, suggests an activation of microglia in the hippocampus of mice undergoing CR-induced serial daily torpor.

### Microglia-associated homeostasis and phagocytic gene expression is transiently downregulated in the hippocampus during daily torpor in CR mice

**Figure 5:**
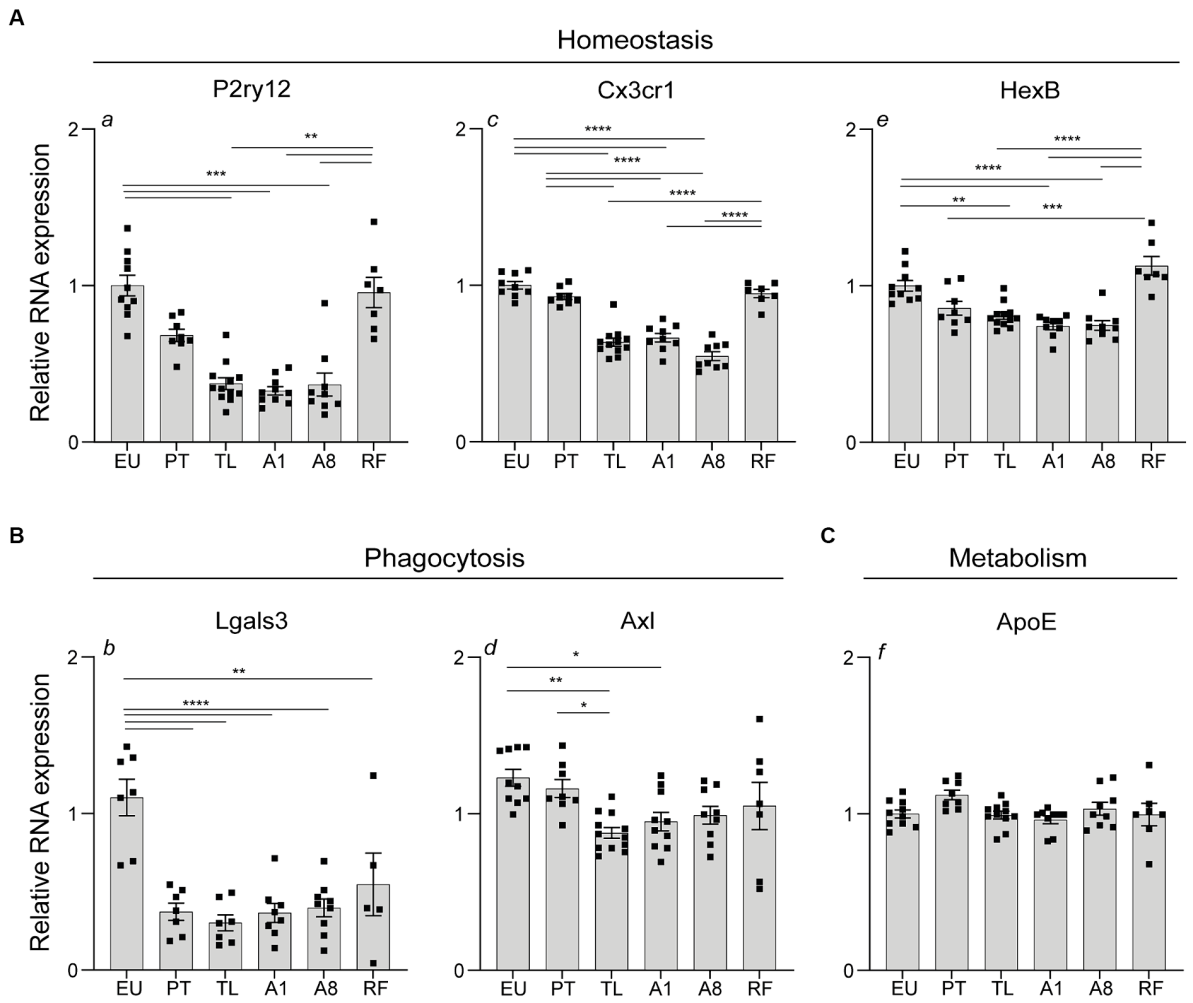
Expression of genes associated with microglia homeostasis, phagocytosis and metabolism in the hippocampus during daily torpor in CR mice. **(A)** Total hippocampal tissue RNA expression of homeostasis (P2ry12, Cx3cr1, HexB), **(B)** phagocytosis (Lgals3, Axl) and **(C)** metabolism-associated (ApoE) genes in control EU mice (not restricted, no torpor), calorie-restricted (CR) mice across torpor (TL) and arousal (A1, A8, PT) phases and refed (RF) mice. RNA expression was normalized to Hmbs and expressed as a fraction of EU animals. EU, euthermia; PT, pre-torpor; TL, torpor late; A1, arousal early; A8, arousal late. Statistical analysis was performed with non-parametric Kruskal-Wallis test with Dunn’s post-hoc comparison (panels a, f) and one-way ANOVA test with Tukey post-hoc comparison (panels b, c, d, e). Data are mean ± SEM; * ≤ 0.05, ** ≤ 0.01, *** ≤ 0.001, **** ≤ 0.0001.

After observing consistent morphological alterations in CA1 microglia, indicative of a shift from their surveillant state, we sought to understand the associated molecular changes. Given that persistent microglia activation often coincides with changes in gene expression, particularly in genes associated with homeostasis, phagocytosis, and lipid metabolism, we examined the mRNA expression of relevant genes in total hippocampal tissue. The mRNA abundance of homeostatic genes *P2ry12* and *Cx3cr1* was significantly lower in TL, A1 and A8 compared to the control EU group but returned to near-control EU levels in PT and RF (Figure 5A). The mRNA levels of microglia homeostatic gene *Hexb*, showed a more subtle decrease in TL, A1 and A8 compared to the control EU group and expression levels returned back to control EU levels in RF (Figure 5A). The expression of phagocytic marker *Lgals3* was downregulated throughout torpor and arousal phases compared to EU and remained lower also in RF, while the expression of *Axl*, another phagocytic marker, decreased modestly in TL and A1 and normalized in PT (Figure 5B). Expression of *Apoe*, a gene involved in lipid metabolism, remained unchanged throughout the different phases of torpor and arousal relative to the control EU group (Figure 5C).

Together, a transient downregulation in the expression of genes related to microglia homeostasis and phagocytosis was observed during torpor and arousal phases A1, A8 in the hippocampus. Expression was largely restored to near-euthermic levels 12 h post-arousal (PT) and when torpor was blocked by refeeding (RF), except for *Lgals3*.

## Discussion

Whereas a few studies have investigated neuroinflammation in hibernators, no study, to our knowledge, has addressed this in daily heterotherms. Using a 30% CR to induce robust serial daily torpor in C57BL/6J mice, our main findings included a transient increase in TNF-α gene expression during torpor and persistent alterations in microglia morphology in the hippocampus.

The use of calorie restriction as a means to induce serial daily torpor in mice introduces two variables that may influence changes in the brain, i.e., CR and torpor. Many benefits of CR including increased lifespan, improved metabolism, enhanced stress response and mitigation of neuroinflammation and neurodegeneration (Mattson, 2012), are also observed in hibernators (Dave et al., 2012), suggesting potential overlapping mechanisms between the two. Indeed, previous research has identified similar physiological responses and gene expression changes, primarily involving the metabolism (Walford and Spindler, 1997; Xu et al., 2013). Because CR alone, in the absence of torpor, can alter gene expression in the brain (Lee et al., 2000; Wu et al., 2009), separating the effects of torpor from those of CR is challenging in our model. However, the transient gene expression changes we observed, specific to torpor-arousal phases, suggest that mechanisms beyond mere CR are at play.

Beyond the gene expression changes, we questioned whether the morphological alterations in microglia were attributable to CR or daily torpor. To our knowledge, no studies specifically addressed CR’s impact on basal microglia morphology in mice. However, CR has been shown to partially counteract age-related functional changes of microglia, essentially ‘rejuvenating’ their functions (Olmedillas Del Moral et al., 2020). Moreover, numerous rodent studies demonstrated CR’s role in attenuating microglial activation in the context of neuroinflammation and age-related neuropathologies (Morgan et al., 1999; Lee et al., 2003; Radler et al., 2014; Zarini et al., 2021). In a rat model of traumatic brain injury, CR preserved microglia in a ramified, surveillance-like state throughout the recovery period, averting a shift to a more classically activated phenotype (Loncarevic-Vasiljkovic et al., 2012). Yet, our observations showing a transition from a ramified phenotype to one characterized by reduced cell branching, complexity, and size – features commonly associated with microglia activation (Karperien et al., 2013; Morrison and Filosa, 2013; Baron et al., 2014; Verdonk et al., 2016; van Olst et al., 2018) – suggest that daily torpor, rather than CR, is the primary factor at play. Supporting this, two studies in the Syrian hamster, a deep hibernator, reported similar microglia morphological changes during torpor (Cogut et al., 2018; Leon-Espinosa et al., 2018). However, while the Syrian hamster showed rapid microglia morphological reversal during the euthermic period of interbout arousal, CR mice displayed only marginal reversal during their euthermic phases (A8 and PT). This disparity may stem from the energy constraint in mice undergoing daily torpor. Unlike the Syrian hamster, which consumes hoarded food upon arousal replenishing their energy sources, CR mice remain under constant low energetic supply because of the dietary restriction. Consequently, CR mice might prioritize their limited energy reserves to processes other than the recovery of microglia morphology during arousals. This notion is further supported by the observation that even after 2 days of increased food intake, the microglial morphology in CR mice does not completely restore to control EU levels.

While CR mice displayed microglial morphological changes akin to those in Syrian hamsters, their status as daily heterotherms sets them apart. The sustained “de-ramified” state of microglia in these mice led us to question whether these alterations signify microglia activation in response to the repetitive metabolic stress of daily torpor, potentially leading to adverse neuroinflammatory events, rather than an adaptive response characteristic of hibernators. Although microglia can exhibit various morphologies in response to different stimuli or injuries (Vidal-Itriago et al., 2022), we did not observe the amoeboid form often associated with an advanced activation state seen in chronic neuroinflammation and neurodegenerative disorders (Muzio et al., 2021). In addition, despite the evident downregulation of microglial homeostatic genes, there was no concurrent upregulation of genes related to phagocytosis and lipid metabolism. This pattern is typically observed in disease-associated microglia (DAM) phenotype found in many neuroinflammatory and neurodegenerative conditions (Hickman et al., 2013; Keren-Shaul et al., 2017; Deczkowska et al., 2018). Further, the transient increase in TNF-α mRNA expression, unaccompanied by elevated levels of other pro-inflammatory cytokines, and the absence of reactive astrogliosis, points toward the absence of a deleterious neuroinflammatory response, in contrast to concomitant changes in these parameters in chronic neuroinflammation and neurodegeneration (Hostenbach et al., 2014). Additionally, the stable 4-HNE protein levels during daily torpor suggest an absence of increased lipid peroxidation, a hallmark of oxidative damage (Breitzig et al., 2016), which is closely associated with neuroinflammatory processes (Smith et al., 2022). Together, our findings indicate that CR-induced serial daily torpor in mice does not trigger a deleterious neuroinflammatory response. Rather, the morphological alterations in microglia might represent a unique neuroimmune response tailored to the metabolic and physiological changes of daily torpor in mice, paralleling observations in deep hibernators.

The upregulation of TNF-α mRNA levels during torpor, which later normalizes during euthermic phases, is especially noteworthy. This pattern differs from our observations in the Syrian hamster, where no increase in TNF-α or other pro-inflammatory cytokines mRNA levels was detected in the hippocampus during torpor (Cogut et al., 2018). This discrepancy might be attributed to differences in torpid body temperatures (Tb). In Syrian hamsters, Tb drops to 8°C, resulting in a likely suppression of transcription (van Breukelen and Martin, 2001, 2002), whereas in CR mice, it only declines to around 25°C. Nevertheless, even mild therapeutic hypothermia (30–35°C) has been shown to attenuate TNF-α mRNA expression levels in the brain (Gu et al., 2015; Liu et al., 2016), suggesting that the upregulation of TNF-α in CR torpid mice might play a crucial role in brain adaptation during daily torpor.

TNF-α, despite being a potent pro-inflammatory cytokine (McCoy and Tansey, 2008), also plays a crucial role in neuroprotection (Hattori et al., 1993; Gary et al., 1998; Tamatani et al., 1999) and is implicated in regulating hippocampal synaptic plasticity, particularly in synaptic scaling (Stellwagen and Malenka, 2006). Specifically, during extended periods of reduced neural activity, TNF-α released by glial cells scales synapses up, by increasing excitatory glutamatergic synaptic responses while simultaneously decreasing inhibitory synaptic strength (Stellwagen et al., 2005; Stellwagen and Malenka, 2006; Pribiag and Stellwagen, 2013). In the context of torpor, characterized by reduced neural activity, this mechanism becomes particularly intriguing. Unlike deep hibernators that undergo substantial hippocampal neuronal dendrite retraction and reduction in spine numbers (Popov and Bocharova, 1992; Magarinos et al., 2006; Popov et al., 2007), a study in mice undergoing fasting-induced torpor revealed no signs of such structural reorganization (de Veij Mestdagh et al., 2021). While deep hibernators likely rely on these structural changes for efficient energy conservation and neuronal protection during extended torpor (Carey et al., 2003), daily heterotherms might prioritize functional adaptations such as synaptic scaling over major structural alterations. Nevertheless, the question remains whether the synaptic changes reported in response to torpor in fasted mice are similar to those of CR mice. Further exploration of the neuronal and synaptic adaptations during CR-induced daily torpor in mice and the potential interplay with TNF-α is warranted.

In summary, our results show that CR-induced serial daily torpor in mice triggers changes in hippocampal gene expression and microglial morphology, which are primarily driven by the torpor state, rather than CR. Despite mice being non-obligatory hibernators, the observed changes do not appear to be harmful. Instead, they point toward activation of adaptive neuroimmune responses that may be instrumental in preserving neuronal integrity during serial torpor-arousal cycles, much like natural hibernators. Given this similarity, CR-induced daily torpor in mice offers a valuable and readily accessible model for studying torpor-associated adaptive brain mechanisms. Unraveling these underlying neuroprotective strategies could have a significant impact on humans, particularly in the context of preventing and developing therapeutic approaches for stroke and neurodegenerative diseases.

## Limitations

This study has some limitations. First, we used only male mice, not addressing potential gender differences. Second, relying solely on lipid peroxidation as a marker for oxidative stress may overlook oxidative stress-induced damage to other macromolecules such as DNA and proteins. A broader evaluation of oxidative stress markers is necessary for a definitive conclusion. Third, the use of total hippocampal tissue to analyze microglia-associated gene expression may not provide an accurate representation, given that some of these genes are also expressed in other cell types. However, the genes we examined, such as *P2ry12* and *Cx3cr1*, are predominantly expressed by microglia in the CNS. Future studies would benefit from isolating microglia to more precisely assess gene expression alterations in mice undergoing CR-induced serial daily torpor. Additionally, corroborating our mRNA data by protein expression assessments would offer a more thorough understanding of the neuroimmune changes associated with torpor. Lastly, our torpor induction method introduces two variables that are challenging to separate from the effects of torpor itself: a shift from nocturnal to diurnal activity, which may have various hormonal, physiological, behavioral and molecular implications, and the potential for cold stress due to sub-thermoneutral housing temperatures. These factors should be carefully considered when interpreting our results.

## Data availability statement

The original contributions presented in the study are included in the article/Supplementary material, further inquiries can be directed to the corresponding author.

## Ethics statement

The animal study was approved by Animal Welfare Body (IVD) of the University of Groningen after approval of the competent authority [Dutch Central Committee for Animal Experiments (CCD), permit AVD105002016427]. The study was conducted in accordance with the local legislation and institutional requirements.

## Author contributions

VC: Conceptualization, Data curation, Formal analysis, Investigation, Methodology, Writing – original draft. MG: Data curation, Investigation, Methodology, Resources, Writing – review & editing. AJ: Data curation, Formal analysis, Investigation, Methodology, Writing – review & editing. MS: Formal analysis, Writing – review & editing. RH: Conceptualization, Data curation, Formal analysis, Investigation, Methodology, Software, Supervision, Writing – original draft.
