# Supplementary material for: Hippocampal neuroimmune response in mice undergoing serial daily torpor induced by calorie restriction
Source: Front Neuroanat. 2024 Apr 15;18:1334206. doi: 10.3389/fnana.2024.1334206 (PMC11056553; doi:10.3389/fnana.2024.1334206)
Supplement: Supplementary file 1 [file Table_1.DOCX]

**Supplementary table 1 –** Primers used for qPCR

| Gene | Accession Number | Forward primer 5’-3’ | Reverse primer 5’-3’ |
| --- | --- | --- | --- |
| *Apoe* | NM_000041 | GTTGCTGGTCACATTCCTGG | CTTCAACTCCTTCATGGTCTCG |
| *Axl* | NM_001699 | GTTTGGAGCTGTGATGGAAGGC | CGCTTCACTCAGGAAATCCTCC |
| *Cx3cr1* | NM_009987.4 | CTTGCCTCTGGTGGAGTCTG | GTGAGGTCCTGAGCAGATGG |
| *Gfap* | NM_010277 | CCTTCTGACACGGATTTGGT | TAAGCTAGCCCTGGACATCG |
| *Hexb* | NM_010422.2 | GGGCTTTGGCAGCGATTTT | ACACTTCGACTACTGTGCCC |
| *Hmbs* | BC-003861 | CCGAGCCAAGGACCAGGATA | CTCCTTCCAGGTGCCTCAGA |
| *Il-6* | NM_031168 | ACAACCACGGCCTTCCCTACTT | CACGATTTCCCAGAGAACATGTG |
| *Lgals3* | NM_010705.3 | CAGGATTGTTCTAGATTTCAGGAG | TGTTGTTCTCATTGAAGCGG |
| *P2ry12* | NM_027571.3 | CAACTCACCTTCACCGGCA | GCCTTGAGTGTTTCTGTAGGGT |
| *Tnf-α* | NM_013693 | TCTTCTGTCTACTGAACTTCGG | AAGATGATCTGAGTGTGAGGG |
